# The Biomechanics of Cartilage—An Overview

**DOI:** 10.3390/life11040302

**Published:** 2021-04-01

**Authors:** Joerg Eschweiler, Nils Horn, Bjoern Rath, Marcel Betsch, Alice Baroncini, Markus Tingart, Filippo Migliorini

**Affiliations:** 1 Department of Orthopaedic Surgery, RWTH Aachen University Clinic, Pauwelsstraße 30, 52074 Aachen, Germany; joeschweiler@ukaachen.de (J.E.); nhorn@ukaachen.de (N.H.); alice.baroncini@gmail.com (A.B.); mtingart@ukaachen.de (M.T.); 2 Department of Orthopaedic Surgery, Klinikum Wels-Grieskirchen, 4600 Wels, Austria; Bjoern.Rath@klinikum-wegr.at; 3 Department of Orthopaedic Surgery, Mannheim University Hospital, 68167 Mannheim, Germany; marcel.betsch@gmx.de

**Keywords:** Articular cartilage, viscoelasticity, biomechanics, tissue engineering, osteoarthritis

## Abstract

Articular cartilage (AC) sheathes joint surfaces and minimizes friction in diarthrosis. The resident cell population, chondrocytes, are surrounded by an extracellular matrix and a multitude of proteins, which bestow their unique characteristics. AC is characterized by a zonal composition (superficial (tangential) zone, middle (transitional) zone, deep zone, calcified zone) with different mechanical properties. An overview is given about different testing (load tests) methods as well as different modeling approaches. The widely accepted biomechanical test methods, e.g., the indentation analysis, are summarized and discussed. A description of the biphasic theory is also shown. This is required to understand how interstitial water contributes toward the viscoelastic behavior of AC. Furthermore, a short introduction to a more complex model is given.

## 1. Introduction

Articular cartilage (AC) is a loadbearing soft tissue that overlies the interacting bony surfaces in diarthrodial joints. AC is a dense connective tissue mainly comprised of collagen, proteoglycans, organized in special zones containing special types of cells called articular chondrocytes [1,2]. The biological and mechanical properties of AC are highly complex and vary zonally [3]. AC provides the joint with different biomechanical functions, e.g., wear resistance or shock absorption up to eight times of the bodyweight [4,5].

Conditions affecting AC and the surrounding structures, in particular osteoarthritis (OA), represent some of the most common diseases encountered in the orthopedic practice [6,7,8,9]. OA is a degenerative disease of the entire joint. Besides subchondral bone changes, joint pain, and discomfort, one factor is characterized by a progressive degenerativ erosion of the AC. OA occurs when the cartilage that covers the ends of the bones in joints gradually deteriorates [10]. Furthermore, it is clinically presented as radiographic narrowing of the joint space, debilitating pain and stiffness, and loss of joint function [11].

Given the high compressive joint forces, degeneration of AC leads to joint pain, stiffness, and impaired mobility [12]. Throughout life, AC undergoes no internal remodeling and turnover [13]. This is especially true for chondrocytes, which are committed to replacing matrix macromolecules lost caused by degradation [2]. However, the healing process of AC barely results in a restitution ab integrum: with persistent chondral defects and fibrocartilage being the result [14]. Moreover, aging decreases the ability of chondrocytes to maintain and restore the cartilage matrix, increasing the risk of AC surface degeneration [2,13].

This review was designed to update current evidence concerning the theoretical background of AC biomechanics, discussing developing concepts, and providing new insights and possible applications.

## 2. Anatomy of Hyaline Articular Cartilage

### 2.1. General Composition

From a mechanical point of view, AC is a composite of materials with widely differing biomechanical properties [15,16,17]. AC distributes loads and peak loads to the underlying bone. Because of its elasticity, AC gives rise to deformations, but always returns to its original shape, and it combines a remarkably long life with low friction. With dynamic loads, a kind of pumping effect occurs over. This results in a spread of proteoglycans around the cells. This leads to an increase of the volume and leads additionally to an increase in tension in the collagen fibers, which increases the elasticity of the tissue.

Table 1 shows an overview of the general composition of AC. Approximately 70% to 80% of the AC weight is water [16,18].

**Figure 1:**
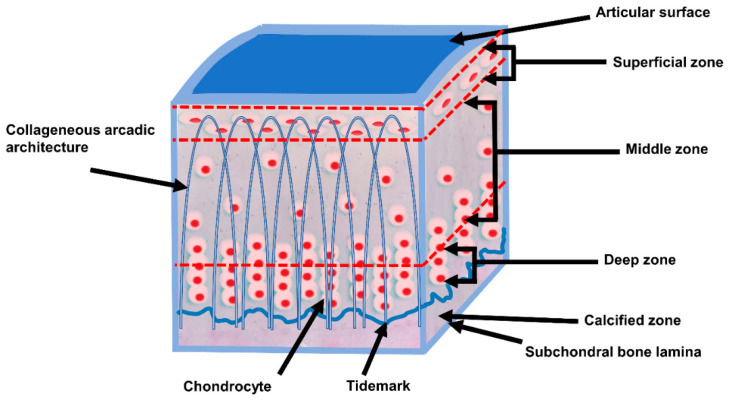
Schematic, cross-sectional diagram of healthy articular cartilage: cellular organization in the zones of articular cartilage (adapted from [24,25]).

Figure 1 shows the schematic composition of AC. Also, the arcade arrangement of the collagen fibers is shown in Figure 1. AC is in the mean 2 to 4 mm thick [19,20] (retropatellar up to 7 to 8 mm [21]), and does not have blood vessels, nerves, or lymphatics [22,23].

### 2.2. Chondrocytes

AC is a tissue composed of a small population of chondrocytes. Chondrocytes are highly specialized and metabolically active, and surrounded by a multicomponent substance called the extra cellular matrix (ECM). The ECM is comprised of water, collagen, and proteoglycans, along with other less common non-collagenous proteins and glycoproteins [22,24,26]. These play an important role in the turnover and repair of the ECM [24]. Chondrocytes originate from mesoderm and vary in shape, number, and size, depending on the zonal regions of the AC [24].

### 2.3. Zonal Regions

AC shows a zonal arrangement (Figure 1 and Table 2). AC is divided into four layers with different types of biomechanical and biochemical behavior.

Proteoglycan concentration and water content vary through the layers of the tissue. In general, the water concentration decreases from superficial to the deep zone, while proteoglycan content increases from the superficial to the deep zone [15,22].

### 2.4. Collagens

Collagen is the most structural macromolecule in AC. It makes up about two-thirds of the dry weight of AC. The resilience of the collagen network depends on the intramolecular and intermolecular connections between the fibrils. Together with the mechanically coupled and water-bounded proteoglycans, the cross-links withstand compressive forces. This will increases the tension in the collagen network. The most important property of collagen fibers is their tensile strength, whereas they will buckle when they will be compressed. The collagen is responsible for the outer shape and the tensile strength of the AC. The water-proteoglycan-compounds ensure the compressive strength, elasticity, and the lifespan of AC.

Type II collagen represents 90% to 95% of the collagen in ECM. Furthermore, collagen types I, IV, V, VI, IX, and XI are involved in building up the framework of fibrils and fibers intertwined with proteoglycan aggregates but contribute only a minor proportion [22,24,28]. The framework and tissue’s matrix, respectively, strength depends on the extensive cross-linking of the collagen fibrils and the apparent zonal changes in fibrillar architecture with tissue depth [22,30]. 

### 2.5. Protein Network

Throughout AC, three macroclasses of proteins can be found: collagens (mostly type II collagen); proteoglycans (primarily aggrecan); and other non-collagenous proteins (including link protein, fibronectin, cartilage oligomeric matrix protein) and the smaller proteoglycans (biglycan, decorin and fibromodulin) [22,26,31,32].

**Figure 2:**
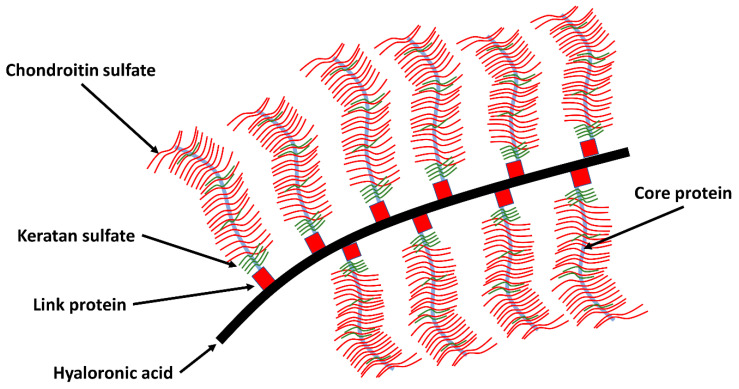
A schematic representation of the collagen network interacting with the proteoglycan network in AC. They have complex macromolecules, also known as aggrecans an elongated protein core to which long glycosaminoglycan chains attach. These carry numerous negative charges and are via chondroitin-4-sulphate, chondroitin-6-sulphate, and keratan sulphate covalently with the aggrecan chain connected. The interstices of this porous solid matrix are filled with water and dissolved ions (adapted to [5,32,33]).

These proteoglycans can bind or aggregate to hyaluronic acid to form a macromolecule (see Figure 2). The network is composed primarily of proteoglycans and collagen. The proteoglycans consist of a core protein to which chondroitin sulfate and keratan sulfate are attached amongst them (Figure 2) [32]. The greater the proteoglycan concentration, the greater the resistance to shear forces. The concentration of proteoglycans is the highest in the middle zone, less in the superficial and deep zone. Proteoglycan and collagen influence each other. Some bridge the distance between collagen fibrils by creating cross connections form and thus make a contribution to the uniform arrangement of the fibrils [22].

## 3. Biomechanics of Articular Cartilage

AC consists of two phases: a fluid phase and a solid phase. Cartilage is seen as a permeable, viscous-elastic material. Its intercellular substance consists mostly of water (up to 65–85%) depending on exposure and previous damage. Water is the principal component of the fluid phase. The solid phase is characterized for most part by the ECM. These boundaries are designed to restrict mechanical deformation. 

**Figure 3:**
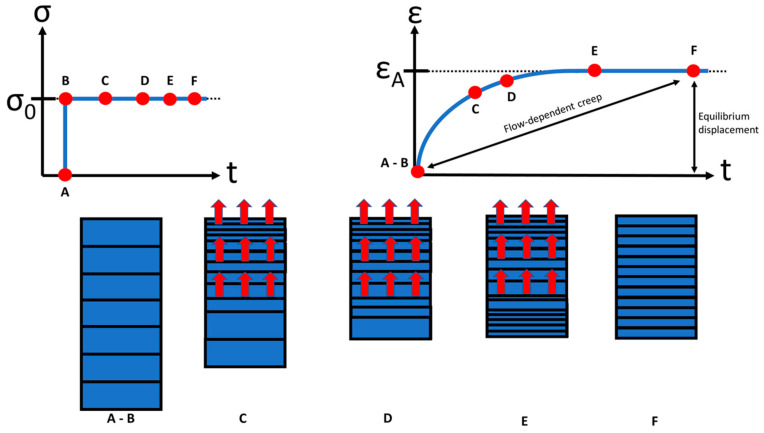
A constant stress σ_0_ applied to a sample of AC (**top left**); creep response ε of the sample under the constant applied stress σ (**top right**). The boxes below the loading and creep curves (A to F) illustrate that creep is accompanied by fluid exudation from the tissue. At equilibrium (t → ∞), fluid flow ceases, and the load is borne entirely by the solid matrix (F). (taken and modified from [35]).

AC and its displacement, respectively, shows a viscoelastic behavior and is a function of time when subjected to a constant load or deformation. Two types of mechanisms are responsible for the viscoelasticity of AC: flow dependent and flow independent [5,23]. The flow-dependent mechanism depends on interstitial fluid. The mechanical response of cartilage is strongly tied to the flow of synovium through the tissue. The displacement of the cartilage is a function of time because the fluid cannot escape from the matrix instantaneously [15] (Figure 3). The application of articular contact forces during joint loading causes an immediate increase in interstitial fluid pressure (Figure 3). If a deformation exists, fluid flows through the cartilage layers and across the articular surface, and causes the fluid to flow out of the ECM. If a pressure difference is applied across a section of cartilage, fluid also flows through the tissue layers. Initially, the displacement is relatively rapid and it corresponds to a more or less large flow of fluid out of the cartilage [2,34]. As the displacement rate slows down and the displacement approaches a constant value, the fluid flow slows in the same way [15]. At equilibrium, the displacement is constant and fluid flow has stopped. 

An application of articular forces during joint loading causes an immediate increase in interstitial fluid pressure which leads to a fluid flow out of the ECM [24]. When the compressive load is removed, interstitial fluid flows back from the inner joint space into the tissue [24]. These observations suggest that cartilage behaves like a sponge, albeit one that does not allow fluid to flow through it easily [15].

The flow-independent component of viscoelasticity is caused by the intrinsic viscoelastic behavior of the collagen-proteoglycan matrix. The pressure of the fluid provides a significant component of total load support, thereby reducing the stress acting upon the solid matrix [24]. Swelling of the aggregated molecules against the collagen framework is an essential element in the mechanical response of cartilage [15]. The relationship between proteoglycan aggregates and interstitial fluid provides compressive resilience to AC through negative electrostatic repulsion forces [24,32]. In an aqueous environment, mutual repulsion of negative charges causes an aggregated proteoglycan molecule to spread out and occupy a large volume [15]. Under compression, the negatively charged sites on aggrecan are pushed closer together. This effect increases their mutual repulsive force, and adds to the compressive stiffness of the cartilage [15,24,25]. If proteoglycans were non-aggregated, they would not be as effective in resisting compressive loads, since they are not as easily trapped in the collagen matrix [15,32].

## 4. Biomechanical Testing of Hyaline Cartilage

To understand the biomechanical behavior and to develop in vitro and in silico models of AC, the mechanical parameters are important to acquire via testing. Beside the mechanical parameters, it has been shown that mechanical stimuli, in the form of compressive or shear loading, can have profound effects on the biosynthetic response of chondrocytes and can serve to impart near-functional properties of the developed tissue constructs [15]. 

**Figure 4:**
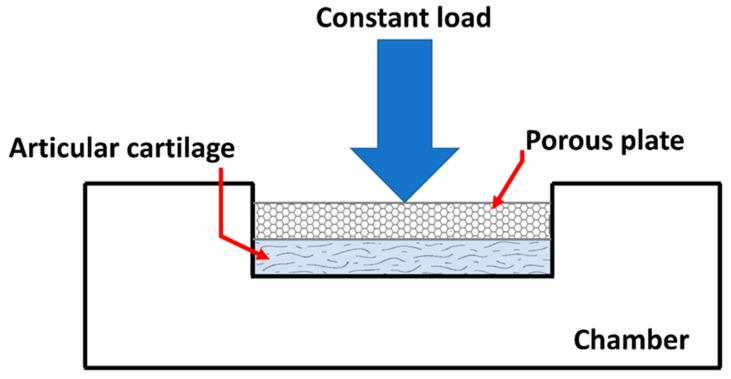
Drawing of an apparatus used to perform a simple compression test of AC. (taken and modified from [15]).

The aim of these test methods is to establish physiological load and boundary conditions, as well as deformation states. A confined compression test is one of the commonly used methods to determine material properties of cartilage (Figure 4). A slice of cartilage is placed in an impervious, fluid-filled well (Figure 4). NaCl-lotion will often be used as a fluid isoton [36].

The tissue is loaded with a constant force throughout the test through a porous plate. Since the well is impervious, flow through the cartilage will only be in the vertical direction and out of the cartilage.

**Figure 5:**
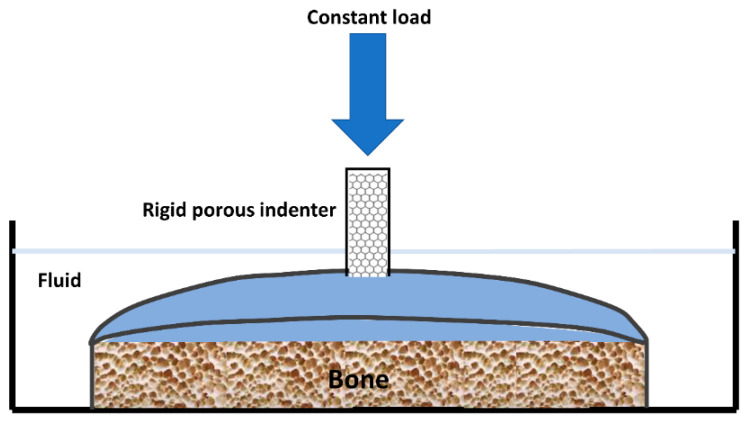
Representation of an apparatus used to perform an indentation test on articular cartilage (taken and modified from [15]).

An indentation test (Figure 5) provides an attractive alternative to confined compression [15]. The cartilage remains attached to its underlying bone, which provides a more natural environment for testing. A constant load is applied to a small area of the cartilage through a porous indenter.

**Figure 6:**
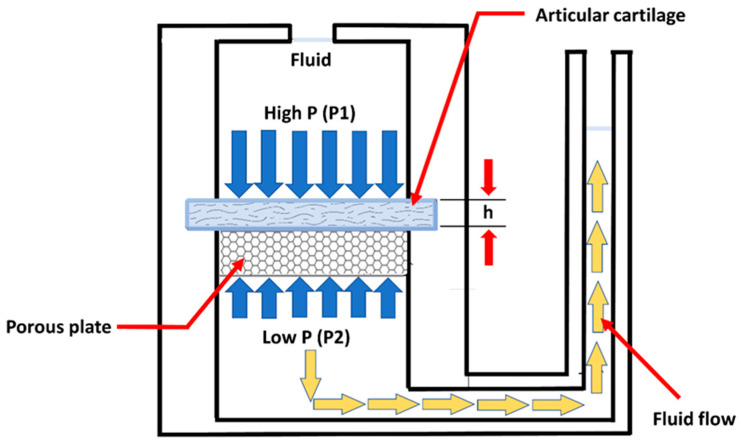
Schematic representation of a device used to measure the permeability of cartilage. A slice of cartilage is supported on a porous plate in a fluid-filled chamber. High pressure applied to one side of the cartilage drives fluid flow. The average fluid velocity through the cartilage is proportional to the pressure gradient, and the constant of proportionality is called the permeability. (taken and modified from [15]).

The permeability of the cartilage is also determined by a confined compression test (Figure 6). The permeability is an index of the resistance to fluid flow through the matrix of the cartilage. The deformation-dependent permeability may be a valuable mechanism to facilitate load sharing between the solid and fluid phases of AC [15].

## 5. Creep and Relaxation of Soft Tissue

**Figure 7:**
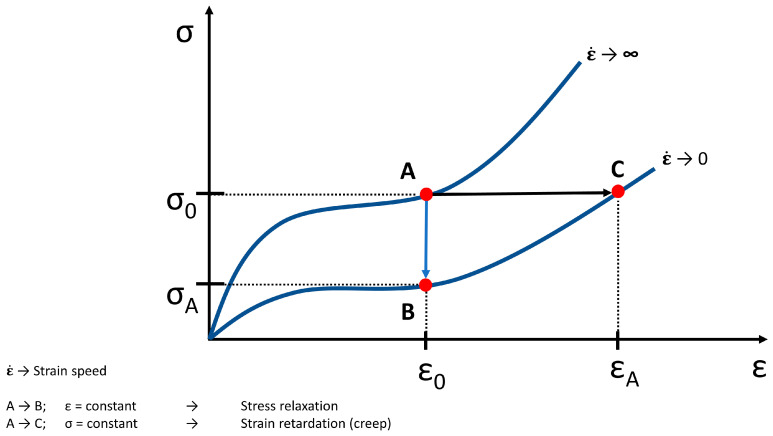
Theoretical tensile loading curves at infinitely high and low strain rates (adapted from [37]), (σ → stress, ε → strain). From A to B the strain is constant (ε_0_) and this means a stress relaxation (σ_A_). From A to C the stress is constant (σ_0_) and this means a strain retardation and creep (ε_A_), respectively. An idealized behavior is shown.

A number of models had been developed to analyze the interrelationship between the elastic and viscous components of tissue biomechanical behavior and the stress-strain behavior of soft tissue, respectively (Figure 7).

**Figure 8:**
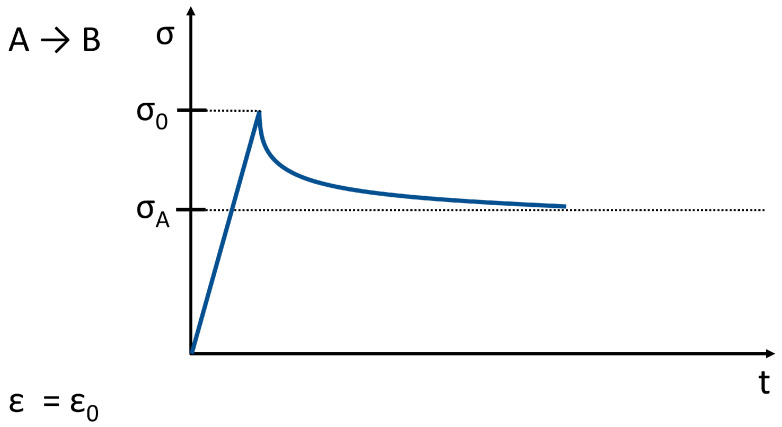
Stress relaxation related to the behavior shown in Figure 7. (σ → stress, ε → strain; the strain is constant; t ≥ 0).

In relaxation mode (Figure 8), a constant displacement is applied to the tissue, and the force needed to maintain the displacement is measured. The deformation results in increased stress and lead to fluid displacement, after which relaxation occurs. The tissue tension as a result of a physiological pressure effect will not hold for a long time because of the relaxation. The result is an increased pressure distribution over a larger area. Most of the joint models work with compressible joint contact surfaces. In developing methods of treating cartilage damage, it is important to take into account that certain physical characteristics of the cartilage does not apply to the entire cartilage surface at the same time.

**Figure 9:**
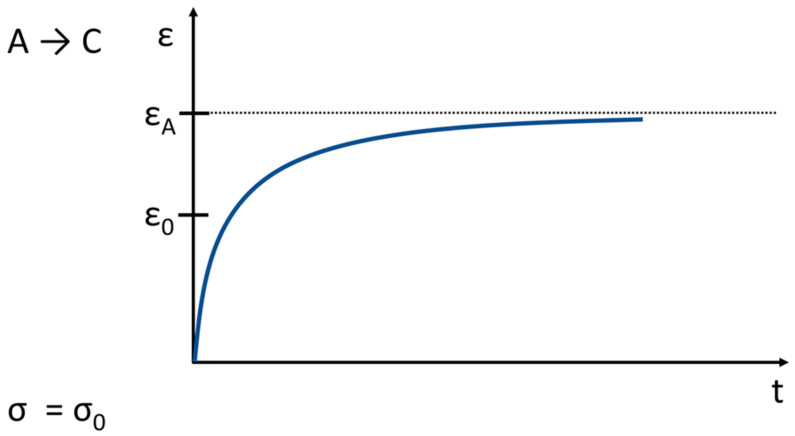
Creep related to the behavior shown in Figure 7. (σ → stress, ε → strain; the stress is constant; t ≥ 0).

In the creep mode (Figure 9), a constant load is applied to the cartilage through a porous plate, and the displacement of the tissue is measured over time. The extent of creep in the cartilage is also determined from the initially strong and later decreasing flow and leakage of the intercellular fluid. The interaction between the surface load and the compressive stress in the collagen-proteoglycan network as well as the friction-related delay in the frictional drag creates an equilibrium, after which the fluid displacement stands still.

The cartilage deforms under a constant load, but the deformation is not instantaneous, as it would be in a single-phase elastic material such as a spring.

## 6. Modeling of Hyaline Articular Cartilage

### 6.1. General Theoretical and Technical Approximation

**Figure 10:**
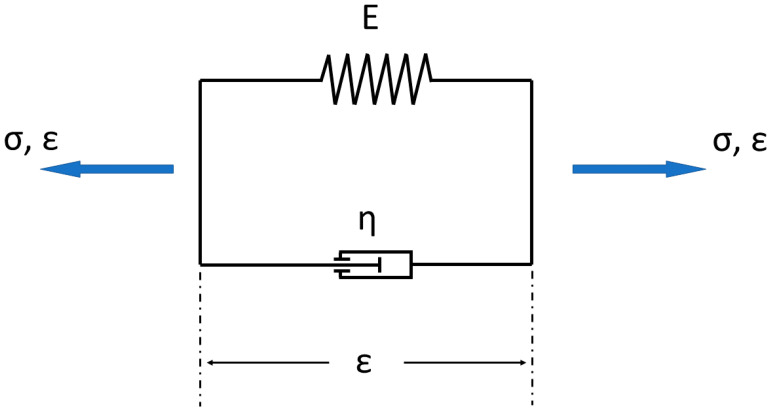
Simple model consisting of a mixture of spring and damper for describing viscoelastic behavior (= Kelvin–Voigt model). (σ → stress, ε → strain, E → is a modulus of elasticity; η → is the viscosity).

The biomechanical behavior of Articular cartilage is better understood when the tissue is viewed as a biphasic medium [24]. In general, there exist models to describe viscoelastic behavior built up as a mixture of fluid (damper) and solid (spring) components (e.g., Figure 10). 

In Figure 10 the simple Kelvin–Voigt model is shown. The overall strain *ε* corresponds to the elastic strain *ε_e_* and viscous strain *ε_v_*.

(1) $$\varepsilon =\varepsilon_{e}=\varepsilon_{v}$$

The force equilibrium (kinetic relationship) delivers the stress in the Kelvin–Voigt model:

(2) $$\sigma =\sigma_{e}+\sigma_{v}.$$

The constitutive relationship will be generated from the material equation for a spring:

(3) $$\varepsilon_{e}=\frac{\sigma_{e}}{E}$$

(This corresponds to Hook’s law), and the material context for a damper:

(4) $${\dot{\varepsilon }}_{v}=\frac{\sigma_{v}}{\eta }$$

(This corresponds to Newtonian fluid behavior).

From the force equilibrium, it is possible to create the general equation for the model:

(5) $$\sigma =E\cdot \varepsilon_{e}+\eta \cdot {\dot{\varepsilon }}_{v}=E\cdot \varepsilon +\eta \cdot \dot{\varepsilon }.$$

For a complex tissue like AC, the model is too simple and needs more information to approximate the biomechanical behavior.

### 6.2. Specific Models of Articular Cartilage

To date, the most successful theory for cartilage compressive viscoelastic behaviors is the **biphasic poroelastic theory** developed by Mow et al. [38]. This biphasic theory models AC as composite materials consisting of a solid phase (solid like components of the cartilage, proteoglycans, collagen, cells and lipids are lumped together to constitute the solid phase) and a fluid phase (interstitial fluid that is free to move through the matrix) [5]. The porous solid matrix is elastic and permeable to fluid. Three major (internal) forces act within the loaded tissue: (1) the stress developed within the deformed collagen—proteoglycan macromolecules (solid) matrix; (2) the pressure that is developed within the fluid phase, furthermore, (3) the frictional drag acting between the fluid phase and the solid phase as they flow past each other [5]. In a biphasic medium, all of the three internal forces act in concert to balance applied external forces, thus giving rise to a viscoelastic effect [5].

By fitting the mathematical biphasic model to the measured displacement, two material properties of the cartilage are determined: the aggregate modulus and permeability [15]. In cartilage biomechanics, instead of Young’s modulus, the aggregate modulus is often used to describe the tissue, because it can be directly calculated from the equilibrium data in a confined compression test (e.g., Figure 4) [5]. The aggregate modulus is a measurement of the stiffness of the tissue at the equilibrium when all fluid flow has slowed down. The higher the aggregate modulus, the less the tissue deforms under a given load [15]. The aggregate modulus of cartilage is typically in the range of 0.5 to 0.9 MPa [15,39].

In addition to the aggregate modulus, the hydraulic permeability k of the cartilage is also determined from a stress-relaxation or creep test (Figure 7, Figure 8 and Figure 9) of confined or unconfined compression. It can be determined by curve-fitting the creep or relaxation curve generated in the test [5,17,40,41]. The permeability k indicates the resistance to fluid flow through the cartilage (solid) matrix. 

In the simplest version of the biphasic theory, the stress—strain law for the solid matrix, is assumed to be isotropic and linearly elastic [5]. The frictional drag acting on the solid phase can be given by Darcy’s law [38]:

(6) $$v_{ave}=k\cdot \nabla p.$$

This means that the average fluid velocity *v_ave_* through a soil sample is proportional to the pressure gradient ∇p and permeability *k*. 

The pressure gradient is approximated by (Figure 9):

(7) $$\nabla p=\frac{p_{1}-p_{2}}{h}.$$

Permeability is not constant through and also varies with deformation of the tissue. The permeability of articular cartilage is highest inching the joint surface and lowest getting closer to the deep zone [42,43,44,45,46]. The permeability influences also the deformation rate. If *k* is high, the fluid can flow out of the matrix easily, and the equilibrium is quickly reached. A lower value for k causes a slower transition from the rapid early displacement to the equilibrium. As cartilage is compressed, its permeability decreases. Under increasing load, the fluid flow will decrease because of the decrease in permeability which accompanies compression [15]. These qualitative results are helpful for interpreting data from tests of physiological and OA related cartilage [15].

Lai et al. [36]. published the **triphasic cartilage modeling framework**, expanding the generalized biphasic models by incorporating an ionic phase [36]. They described how swelling pressure, Donnan osmotic pressure, and solid matrix stress are related to one another.

A distinction is made between three phases in cartilage: a solid phase with a tightly packed collagen network and a high concentration of proteoglycan aggregates, a liquid phase with a high water content and a ion phase in which the environment to ensure electrical neutrality is dominated by dissolved electrolytes (anions, cations).

The physico-chemical view of cartilage and other hydrated charged tissue are based on the Donnan theory for aqueous polyelectrolyte solutions [47]. The Donnan equilibrium and Gibbs–Donnan equilibrium, respectively, describes the equilibrium between two solutions that are separated by a membrane. The membrane allows a passage of different charged ions of the solutions, and is thus a selectively permeable membrane. The effect is related to the presence of impermeant ions, which means that they are unable to pass through a semipermeable membrane upon one side of a boundary on the distribution of permeant ions across the boundary. The membrane permeability and impermeability, respectively, is related to the size of the particular ion, which can be too large to pass through the membrane pores from one to the other side. In the case that the concentration of those ions passing freely, the membrane is equal the total number of charged molecules on either side of the membrane is equal [36]. In the case that there exists a selective permeability of the membrane, an electrical potential between the two sides of the membrane will be developed [36]. The result is that the two solutions vary in osmotic pressure. That means one solution have in the end more of a certain type or types of ion that does the other solution [47]. 

According to this model, cartilage is partly a porous but solid material and partly a non-compressible liquid. The pore diameter is between 2.5 nm and 6.5 nm [38], its permeability (total weight minus dry weight/total weight) is between 60% and 85%. The micropores make sure that molecules only can pass up to a size of 20,000 daltons. There exist a selection process according to spatial molecular properties. When compressed, the diameter increases the pores, which reduces the permeability of the cartilage.

According to biphasic and triphasic theories, equilibrium modulus of cartilage includes the contributions from two sources: the Donnan osmotic pressure and the ‘‘intrinsic stiffness’’ of the solid matrix without charge effect [5]. The apparent and intrinsic tissue properties are defined as the tissue properties with and without the osmotic pressure effects—that is, the properties in triphasic framework as intrinsic properties and those in biphasic model as apparent properties [5,23]. The triphasic mixture theory has been successfully used to describe the flow-dependent and flow-independent viscoelastic behaviors, swelling behaviors, and electro-kinetic behaviors of charged, hydrated AC [5].

An overview about further and more complex models is given in [23]. 

## 7. Conclusions

AC is a highly specialized and complex connective tissue which provides a smooth, lubricated surface for articulation. Normal synovial joints operate with a low coefficient of friction, about 0.001 because of the biomechanical behavior of AC. It facilitates load transmission, with a low frictional coefficient. The coefficient of friction is three times lower than ice on ice [48]. Progressive degeneration of AC leads to dysfunction coming along with OA.

The interaction of the fluid and solid components constitute the mechanical behavior of AC. Significant advances have been made in the last decade in experimental, theoretical, and biological studies of the basic sciences relating to AC [5]. Towards innovative approaches in testing AC, new information can be obtained, and AC can be studied in greater detail in terms of its nonlinear and viscoelastic behaviors, depth-dependent inhomogeneity of the mechano electrochemical properties, and anisotropy characteristics [5]. Furthermore, advanced theoretical models were built up based on the results of more complex experimental tests.

To date, however, there is little research on behavior of cartilage as part of a tissue system and even less as an influence, which the synovia and subchondral bone have on them. Also little research has been done on differences between cartilage cells. The research mostly deals with effects of stress and relief while given in normal cartilage always stress is still there. The effects of stress changes actually represents a better expectation of the biomechanical behavior of the chondrocyte.

The unique structure of AC continues to make its treatment and repair a significant challenge [24]. Investigations regarding the pathogenesis of OA, the form of OA that develops following trauma, help to explain the development and progression of joint degeneration (Buckwalter et al., 2005). The simultaneously measured mechanical and biochemical properties will inevitably provide significant information toward the understanding of OA etiology, especially during the early stages of the disease process [5]. 

## Figures and Tables

**Table 1:** General composition of Articular cartilage (AC) (modified from [16,18]).

**Chondrocytes**	1–10%
**Water**	70–80%
**Collagen**	12–14%
- Type II	10–12%
- Type IX	~1%
- Type XI	~1%
**Proteoglycans**	7–9%
- Hyaluronic acid—proteoglycan—aggregates	6–8%
- Other proteoglycans	~1%
**Mineralic materials**	<4%
**Matrix proteins**	<1%

**Table 2:** Zonal regions of AC (according to [2,15,22,24]).

Zone	Name	Description	Functional Behaviour
**Zone I**	**superficial (tangential) zone**	The superficial zone makes up approximately 10% to 20% of AC thickness. The collagen fibers of this zone are packed tightly and aligned parallel to the articular surface. The chondrocytes in the superficial zone are flatter and smaller and generally have a greater density than that of the cells deeper in the matrix. It protects deeper layers from shear stresses. The zona superficialis has most of the water and very little proteoglycans. In general, the cells are not very active, which means that there is little wear and tear. The zone can also be used as a barrier against large molecules, for example antibodies.	The superficial zona is responsible for the behavior of the cartilage under stress. It deforms more strongly and is therefore less rigid than the underlying zones (Guili.k et al., 1995b). If this zone is disturbed tissue permeability increases, leading to greater fluid exchange of the cartilage with its surroundings and during compression, this leads to greater mechanical stress on the macromolecular network.
**Zone II**	**middle (transitional) zone,**	The middle zone represents 40% to 60% of the total AC volume, and it contains proteoglycans and thicker collagen fibrils. In this layer, the collagen is organized obliquely. Chondrocytes are spherical and at low density.	Functionally, the middle zone is the first line of resistance against compressive forces.
**Zone III**	**deep zone**	The deep zone represents approximately 30% of the AC volume. The deep zone contains the largest diameter collagen fibrils in a radial disposition, the highest proteoglycan content, and the lowest water concentration. The chondrocytes are typically arranged in a columnar orientation, parallel to the collagen fibers and perpendicular to the joint line.	It is responsible for providing the greatest resistance to compressive forces, given that collagen fibrils are arranged perpendicular to the articular surface. This creates a arcade formation. These arcades are to be created by the attempt to transfer the initial fibril network to a higher order, whereby the arcadic structure supports the overlying load.
**Interface**	**tide mark**	The tide mark distinguishes the deep zone from the calcified cartilage. This irregularly salty layer lies between the Zonae radiata and calcificata and separates the lime-poor from the lime-rich cartilage. O’Connor describes this area as the mineralization front of the cartilage [27]. According to Mankin, this layer arises by causing the collagen bundles to twist before going deeper to penetrate the zona calcificata and the bone tissue [28].	It consists of a band of fibrils attached to the collagen fibers which are anchored in the lime-poor layer, and thus prevent them from tearing off cartilage from bone [29].
**Zone IV**	**calcified zone**	The calcified zone plays an integral role in securing the cartilage to bone by anchoring the collagen fibrils of the deep zone to subchondral bone. In this zone, the cell population is scarce and chondrocytes are hypertrophic.	The calcified zone has numerous protrusions, hollows, and interlacing, which gives an excellent resistance to shear forces to prevent the cartilage detaching from the underlying bone.

## Data Availability

Not applicable.
